# The effects of manganese oxide octahedral molecular sieve chitosan microspheres on sludge bacterial community structures during sewage biological treatment

**DOI:** 10.1038/srep37518

**Published:** 2016-11-21

**Authors:** Fei Pan, Wen Liu, Yang Yu, Xianze Yin, Qingrong Wang, Ziyan Zheng, Min Wu, Dongye Zhao, Qiu Zhang, Xiaoman Lei, Dongsheng Xia

**Affiliations:** 1 School of Environmental Engineering, Wuhan Textile University, Wuhan, 430073, China; 2 Engineering Research Centre for Clean Production of Textile Dyeing and Printing, Ministry of Education, Wuhan Textile University, Wuhan, 430073, China; 3 Environmental Engineering Program, Department of Civil Engineering, Auburn University, Auburn, AL 36849, USA; 4 School of Materials Science and Engineering, Wuhan Textile University, Wuhan 430073, China

## Abstract

This study examines the effects of manganese oxide octahedral molecular sieve chitosan microspheres (Fe_3_O_4_@OMS-2@CTS) on anaerobic and aerobic microbial communities during sewage biological treatment. The addition of Fe_3_O_4_@OMS-2@CTS (0.25 g/L) resulted in enhanced levels of operational performance for decolourization dye X-3B. However, degradation dye X-3B inhibition in the presence of Fe_3_O_4_@OMS-2@CTS was recorded as greater than or equal to 1.00 g/L. Illumina MiSeq high throughput sequencing of the 16 S rRNA gene showed that 108 genera were observed during the anaerobic process, while only 71 genera were observed during the aerobic process. The largest genera (*Aequorivita*) decreased from 21.14% to 12.65% and the *Pseudomonas* genera increased from 10.57% to 12.96% according to the abundance in the presence of 0.25 g/L Fe_3_O_4_@OMS-2@CTS during the anaerobic process. The largest *Gemmatimonas* genera decreased from 21.46% to 11.68% and the *Isosphaerae* genera increased from 5.8% to 11.98% according to the abundance in the presence of 0.25 g/L Fe_3_O_4_@OMS-2@CTS during the aerobic process. Moreover, the X-ray photoelectron spectroscopy results show that the valence states of Mn and Fe in Fe_3_O_4_@OMS-2@CTS changed during sewage biological treatment.

Metal oxide nanoparticles (NPs) have been widely applied in various industries (e.g., catalysis^1^, medicine^2^ and water/wastewater treatment^3^) and are among the most commonly used nanomaterials^4^. The excellent physiochemical properties of metallic/metal oxide NPs can be attributed to their high ratio of surface area to volume^5^. However, more metallic/metal oxide NPs are likely to accumulate in atmospheric, soil, or water environments. The toxic properties of metallic/metal oxide NPs can have adverse effects on human health and the environment^6^.

Metallic/metal oxide NPs can enter wastewater treatment plants (WWTPs) through discharge from various consumer products. Biological wastewater treatment methods have been employed for more than one hundred years^7^, and to date, they still maintain a prominent role in the environmental protection., Numerous bioreactor configurations had been operated for wastewater treatments, such as the widely used upflow anaerobic sludge blanket (UASB) and sequencing batch reactor (SBR)^8^. Both UASB and SBR had been successfully employed in wastewater treatment on many municipal and industrial fields for several decades^9^. Metallic/metal oxide NPs can have toxic effects on microorganisms within these biological wastewater treatment systems^10^. *Photobacterium phosphoreum*, a representative bacterium in aquatic environments, is inhibited by metallic/metal oxide NPs (e.g., Ag, TiO_2_, Fe_3_O_4_ and CeO_2_)^11^,^12^. Metallic/metal oxide NPs, namely, ZnO, Fe_2_O_3_, NiO, Cr_2_O_3_ and Co_3_O_4_, can affect microorganisms^13^,^14^. Antimicrobial mechanisms of the phototoxicity of nano-TiO_2_ involve nano-TiO_2_/bacteria surface interactions, the aggregation of nano-TiO_2_, and intrinsic photoactivity^15^.

Octahedral molecular sieve (OMS-2) NPs have extensive applications^5^ and present excellent organic dye decolourization capabilities in the presence of peroxymonosulfate (PMS)^16^. Unfortunately, OMS-2 catalysts cannot yet be applied, as it is difficult to recycle OMS-2 catalysts from solutions. Chitosan (CTS) is widely applied, as it is broadly available and inexpensive^17^,^18^. Currently, magnetic chitosan microspheres, which include combinations of magnetic nanoparticles with CTS, are of particular interest with respect to applications, as they are highly effective at separating and protecting magnetic nanoparticles from agglomeration^19^. However, the potential environmental impacts of manganese oxide octahedral molecular sieve chitosan microspheres (Fe_3_O_4_@OMS-2@CTS) are unknown. High-throughput sequencing methods (e.g., Illumina MiSeq high throughput sequencing) can be used to detect detailed information about microbial communities^20^. However, few studies have been conducted on the effects of OMS-2 NPs on efficiency and microbial communities of typical biological processes (UASB and SBR) during sewage treatment.

In this study, we examine the effects of Fe_3_O_4_@OMS-2@CTS on anaerobic and aerobic microbial communities during sewage biological treatment. First, the catalytic degradation of reactive brilliant red X-3B by Fe_3_O_4_@OMS-2@CTS was studied. Then, Fe_3_O_4_@OMS-2@CTS was added to anaerobic and aerobic sewage biological reactors to assess the degradation of reactive brilliant red X-3B. The anaerobic sewage biological reactor that was used was an upflow anaerobic sludge blanket (UASB), and the aerobic sewage biological reactor that was used had a sequencing batch reactor activated sludge process (SBR) mechanism. We applied the Illumina MiSeq sequencing technique to analyse the effects of NPs on anaerobic and aerobic microorganisms. Our observations serve as key data on the application of NPs in WWTPs.

## Results and Discussion

### Degradation of reactive brilliant red X-3B by Fe_3_O_4_@OMS-2@CTS /PMS during sewage biological treatment

**Figure 1:**
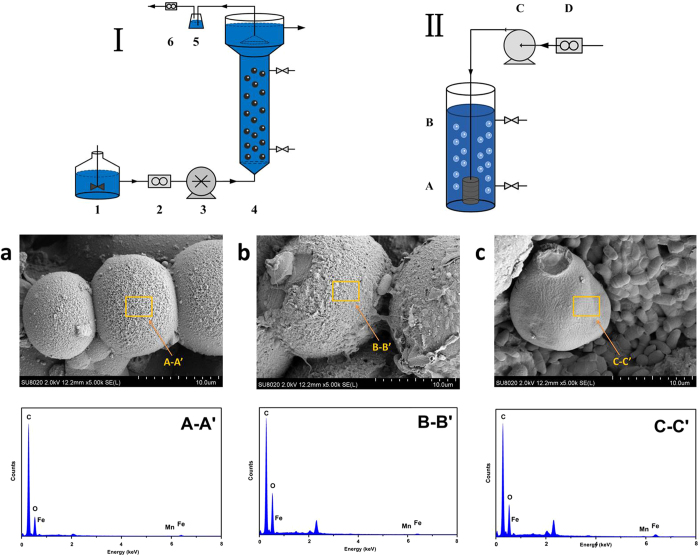
The first line:Schematic diagram of the upflow anaerobic sludge blanket (UASB) reactor device (I) and sequencing batch reactor activated sludge process (SBR) reactor device (II). (I): **1** feed tank, **2** flow counter, **3** peristaltic pump, **4** reactor, **5** water-sealed bottle, and **6** wet gas flow metre (II): **A** aeration device, **B** reactor, **C** gas pump, **D** flow counter. The second line: **(a)** SEM image of fresh Fe_3_O_4_@OMS-2@CTS, **(b)** SEM image of used Fe_3_O_4_@OMS-2@CTS in UASB reactors, **(c)** SEM image of used Fe_3_O_4_@OMS-2@CTS in SBR reactors. The third line: **A–A′.** EDS spectra of fresh Fe_3_O_4_@OMS-2@CTS, **B–B′**. EDS spectra of used Fe_3_O_4_@OMS-2@CTS in UASB reactors, **C–C′.** EDS spectra of used Fe_3_O_4_@OMS-2@CTS in SBR reactors.

Liquid-phase reactions may be an efficient way to recover and recycle magnetic beads^16^. The magnetization curves of Fe_3_O_4_@OMS-2@CTS were measured, and the results are shown in Fig. S1 (Supplementary Information). The magnetization saturation value of pure Fe_3_O_4_ was measured at 107 emu/g, while that of Fe_3_O_4_@OMS-2@CTS was measured at approximately 18 emu/g. The SEM of Fe_3_O_4_@OMS-2@CTS is demonstrated in Fig. 1a (fresh material). The surface morphology of Fe_3_O_4_@OMS-2@CTS is clearly shown in Fig. S2 (a) of the Supplementary Information. A microporous and fibrous structure can be observed through section 1–1′ in Fig. S2 (1–1′). Figure 1b and c shows that the Fe_3_O_4_@OMS-2@CTS remained in the UASB reactor for 60 d and the SBR reactor for 60 d, respectively. In addition, EDS of Fe_3_O_4_@OMS-2@CTS through section A-A′ (Fig. 1 A-A′), B-B′ (Fig. 1 B-B′) and C-C′ (Fig. 1 C-C′) indicate that the main elements of Fe_3_O_4_@OMS-2@CTS are C and O due to the coating of chitosan, while Fe and Mn can also be detected (further confirmed by XPS analysis).

The XRD patterns of Fe_3_O_4_, CTS and Fe_3_O_4_@OMS-2@CTS were presented in Fig. S3 (a) of the Supplementary Information. The results of the FT-IR spectroscopy of OMS-2, Fe_3_O_4_, CTS and Fe_3_O_4_@OMS-2@CTS are shown in Fig. S3(b) of the Supplementary Information. Fe_3_O_4_@OMS-2@CTS exhibits as a core-shell structure, with chitosan acting as the shell and Fe_3_O_4_@OMS-2 as the core. Each component of Fe_3_O_4_@OMS-2@CTS played an important role in X-3B degradation: (1) Fe_3_O_4_ magnetizes the NPs for NP recovery, (2) OMS-2 was the primary catalytic site, which can activate PMS for X-3B degradation^3^, and (3) chitosan can first absorb X-3B onto the material for further degradation by OMS-2. Therefore, removal of X-3B was processed as two steps: X-3B was first absorbed by the microspheres of chitosan and then degraded by OMS-2/PMS. Without OMS-2 and PMS, chitosan can only absorb X-3B, but cannot degrade dyes, which has been confirmed by previous studies^21^,^22^,^23^.

**Figure 2:**
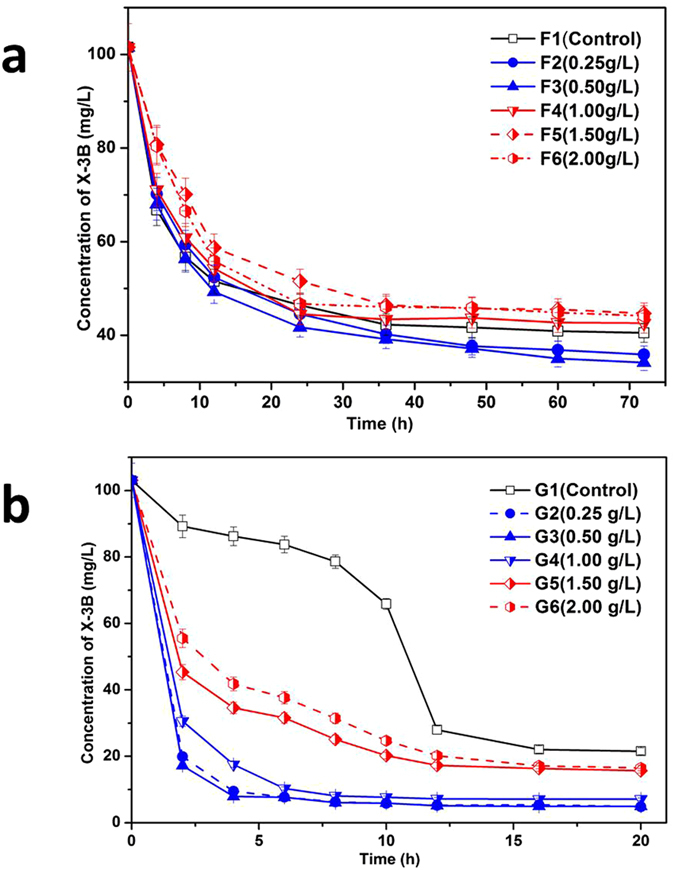
The curve of decolourization ratio in different treatments. (**a**) Change of decolourization ratio in UASB reactors; (**b**) Change of decolourization ratio in SBR reactors.

The COD removal efficiency, decolourization ratio and COD balance levels of the UASB and SBR reactors are summarized in Table 1 for the steady operation stage. The data shown in Table 1 indicate that the UASB and SBR reactors were in good condition for dyeing wastewater processing. Although COD removal efficiencies were similar after addition of Fe_3_O_4_@OMS-2@CTS to both UASB and SBR, we found significantly different results on the decolourization ratio after adding Fe_3_O_4_@OMS-2@CTS to the UASB and SBR reactors. Figure 2a shows that the decolourization ratio in the UASB reactors could be enhanced at low concentrations of Fe_3_O_4_@OMS-2 @CTS (no more than 0.50 g/L), while the decolourization ratio can inhibit high concentrations of Fe_3_O_4_@OMS-2 @CTS (more than 0.50 g/L but no more than 2.00 g/L). The decolourization ratio in the UASB reactors was similar at concentrations of 0.25 g/L and 0.50 g/L, and the decolourization ratio in the UASB reactors was similar at concentrations of 1.00 g/L, 1.50 g/L and 0.50 g/L.

However, the decolourization ratio in the SBR reactors would have been enhanced within the same Fe_3_O_4_@OMS-2 @CTS range (from 0.25 g/L to 2.00 g/L). Figure 2b shows that the maximum decolourization ratio occurred at a concentration of 0.50 g/L and that the minimum decolourization ratio occurred at a concentration of 2.00 g/L. All of the decolourization ratios with Fe_3_O_4_@OMS-2 @CTS (from 0.25 g/L to 2.00 g/L) were higher than the control value.

The different performances on decolourization ratio of these two bio-reactors are mainly due to their own characteristics. Both UASB and SBR reactors are important biological wastewater treatment technologies in WWTPs. UASB shows great resistances to treat wastewater containing biotoxicity compounds because of the dense structure of granular sludge^24^. The effect on the substrate in the UASB reactor should be small. However, SBR usually presents dynamic conditions, as the microbes in the reactor consistently vary along with the change of nutrients and essential elements^25^. After the addition of the material, significant enhancement on decolourization performance in SBR results from the following two reasons: (1) the activity of microbes in SBR was greatly enhanced due to the introduction of essential elements (Fe/Mn), and (2) the added material could also remove X-3B. This could explain the decolourization ratio result that indicates that the change of the decolourization ratio in UASB reactor was much less than that in SBR reactor with the same dosage of Fe_3_O_4_@OMS-2@CTS in both UASB and SBR reactors.

### MiSeq-pyrosequencing results and microbial community structures

Figure 2 shows the results of the decolourization ratio, which indicated significant change in the different treatments. In UASB reactors, the decolourization ratios of F2 and F3 were similar, while the decolourization ratios of F4, F5 and F6 were similar. In SBR reactors, the decolourization ratios of G2, G3 and G4 were similar, while the decolourization ratios of G5 and G6 were similar. According to the decolourization ratio that was effected by the material dosage, samples collected from anaerobic (UASB-F1, UASB-F2 and UASB-F6) and aerobic reactors (SBR-G1, SBR-G2 and SBR-G6) were subjected to Illumina pyrosequencing.

Sequence information on the 6 samples is listed in Table 2. The Chao1/ace estimator and Simpson/Shannon diversity level are widely used microbial diversity indexes that reflect microbial phylotype richness levels and are calculated using a software mothur (http://www.mothur.org/) based on the sequence information. It is evident that Chao1 and Ace in the UASB were different from Chao1 and Ace in the SBR, demonstrating that Chao1 and Ace values of low concentration levels (0.25 g/L) of Fe_3_O_4_@OMS-2@CTS were higher than the control in the UASB and that the Chao1 and Ace of high concentrations (2.0 g/L) of Fe_3_O_4_@OMS-2@CTS were lower than the control. Both low (0.25 g/L) and high concentration (2.0 g/L) Fe_3_O_4_@OMS-2@CTS of the Chao1 and Ace were higher than the control in the SBR.

The behaviours and functions of bacteria may influence microbial activity and biomass, and rarefaction analyses can be used to compare and standardize the detected taxon richness levels between samples^26^. The rarefaction curves of samples in the UASB and SBR reactors are illustrated in Fig. S4 (Supplementary Information). We found similar patterns for the 3 samples in the UASB reactors and for the 3 samples in the SBR reactors. A Venn diagram that can be used to evaluate the distributions is presented in Fig. S5 (Supplementary Information). From the Venn diagram of samples in the UASB reactors, 1,149 OTU species occupied group UASB-F1 (Control), 1,048 OTU species occupied group UASB-F2 (0.25 g/L), and 1,163 OTU species occupied group UASB-F6 (2.0 g/L). The total richness level for all of the groups was measured at 1,321 OTUs. The Venn diagram shows that 820 OTUs containing 62.07% of the sequences were common among all of the samples in the UASB reactors.

The Venn diagram of the samples in the SBR reactors shows that 660 OUT species occupied group SBR-G1 (Control), 641 OTU species occupied group SBR-G2 (0.25 g/L), and 642 OTU species occupied group SBR-G6 (2.0 g/L). The total richness of all of the groups was 803 OTUs. The Venn diagram shows that 446 OTUs containing 54.79% of the sequences were common among all samples in the SBR reactors.

### Taxonomic complexities of bacterial communities

**Figure 3:**
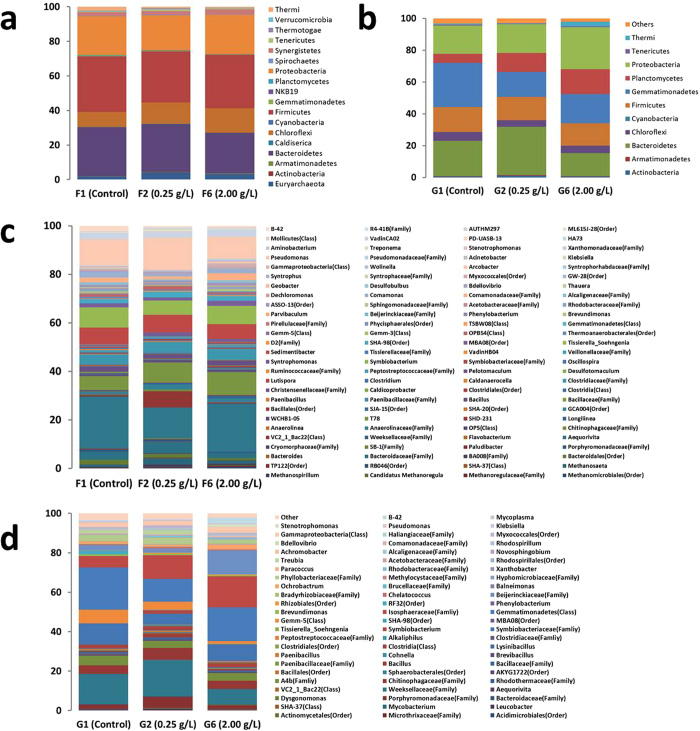
Relative read abundance of different bacterial community structure at phylum and genus levels in different treatments. (**a**) Distribution of bacterial phyla (abundance ≥ 0.1%) in F1, F2 and F6 UASB reactors; (**b**) Distribution of bacterial phyla (abundance ≥ 0.1%) in G1, G2 and G6 SBR reactors; (**c**) Distribution of bacterial genera (abundance ≥ 0.1%) in F1, F2 and F6 UASB reactors; and (**d**) Distribution of bacterial genera (abundance ≥ 0.1%) in G1, G2 and G6 SBR reactors.

There were different abundance levels of bacteria associated with the 3 UASB reactors and 3 SBR reactors. The bacteria community abundance levels of the 3 UASB reactors and 3 SBR reactors at the phylum level are illustrated in Fig. 3a,b. We found 18 identified bacteria phyla from the 3 UASB reactors and 11 identified bacteria phyla from the 3 SBR reactors. *Firmicutes*, *Bacteroidetes*, *Proteobacteria* and *Chloroflexi* were the dominant phyla in bacteria from the 3 UASB reactors, and a change in the 4 dominant phyla was evident in the 3 UASB reactors. The *Firmicutes* percentages in UASB-F1 (Control), UASB-F2 (0.25 g/L) and UASB-F6 (2.0 g/L) were measured at 32.27%, 29.53% and 30.76%, respectively. Percentages of *Bacteroidetes* in UASB-F1 (Control), UASB-F2 (0.25 g/L) and UASB-F6 (2.0 g/L) were recorded at 28.37%, 27.73% and 23.55%, respectively. Percentages of *Proteobacteria* in UASB-F1 (Control), UASB-F2 (0.25 g/L) and UASB-F6 (2.0 g/L) were measured at 28.37%, 27.73% and 23.55%, respectively. Percentages of *Chloroflexi* in UASB-F1 (Control), UASB-F2 (0.25 g/L) and UASB-F6 (2.0 g/L) were measured at 8.51%, 12.41% and 14.23%, respectively. Thus, *Firmicutes*, *Bacteroidetes*, and *Proteobacteria* were weakened, while *Chloroflexi* was enriched during the Fe_3_O_4_@OMS-2@CTS treatments. *Chloroflexi* phylum is also a filamentous bacterium, thus potentially enhancing the anaerobic degradation of wastewater in a UASB^27^.

*Gemmatimonadetes*, *Bacteroidetes*, and *Proteobacteria* were the dominant bacteria phyla from the 3 SBR reactors, and changes in the 3 dominant phyla were also evident for the 3 SBR reactors. The percentages of *Gemmatimonadetes* in SBR-G1 (Control), SBR-G2 (0.25 g/L) and SBR-G6 (2.0 g/L) were measured at 27.83%, 15.62% and 18.28%, respectively. The percentages of *Bacteroidetes* in SBR-G1 (Control), SBR-G2 (0.25 g/L) and SBR-G6 (2.0 g/L) were measured at 22.22%, 30.33% and 14.48%, respectively. The percentages of *Proteobacteria* in SBR-G1 (Control), SBR-G2 (0.25 g/L) and SBR-G6 (2.0 g/L) were measured at 17.72%, 18.12% and 26.37%, respectively; *Gemmatimonadetes* was weakened, while *Proteobacteria* was enriched during the Fe_3_O_4_@OMS-2@CTS treatments. *Bacteroidetes* was enriched in low concentrations and was weakened in high concentrations of Fe_3_O_4_@OMS-2 @CTS treatments. It has been reported that an increase in *Bacteroidetes* in microbiota is correlated with the alleviation of metabolic disorders, as it promotes microbial metabolism^28^. *Proteobacteria* was used as a sign of dysbiosis in bacterial communities^29^. Both *Bacteroidetes* and *Proteobacteria* could enhance biological removal of organic pollutants during SBR^30^.

The bacterial community abundance is analysed further at the genera level in Fig. 3c,d. We identified 108 genera in the bacteria from the 3 UASB reactors and 71 genera in the bacteria from the 3 SBR reactors. *Pseudomonas*, *Aequorivita*, *Tissierella_Soehngenia, Sedimentibacter* and *T78* were the dominant phyla in the bacteria from the 3 UASB reactors, and a change in 5 dominant genera was found in the 3 UASB reactors.

The percentages of *Pseudomonas* in UASB-F1 (Control), UASB-F2 (0.25 g/L) and UASB-F6 (2.0 g/L) were measured at 10.57%, 12.96% and 9.06%, respectively. The percentages of *Aequorivita* in UASB-F1 (Control), UASB-F2 (0.25 g/L) and UASB-F6 (2.0 g/L) were measured at 21.14%, 12.65% and 19.55%, respectively. *Pseudomonas* and *Aequorivita* genera were also found in membrane bioreactors (MBRs)^31^. The percentages of *Tissierella_Soehngenia* in UASB-F1 (Control), UASB-F2 (0.25 g/L) and UASB-F6 (2.0 g/L) were measured at 8.23%, 5.92% and 7.64%, respectively. The percentages of *Sedimentibacter* in UASB-F1 (Control), UASB-F2 (0.25 g/L) and UASB-F6 (2.0 g/L) were measured at 7.01%, 7.35% and 6.11%. The percentages of *T78* in UASB-F1 (Control), UASB-F2 (0.25 g/L) and UASB-F6 (2.0 g/L) were measured at 5.79%, 8.37% and 9.47%, respectively. Thus, *Pseudomonas*, *Sedimentibacter, Tissierella_Soehngenia* and *Aequorivita* were weakened in the Fe_3_O_4_@ OMS-2@CTS treatments, while *T78* was enriched in the Fe_3_O_4_@ OMS-2@CTS treatments.

*Gemmatimonadetes* (Class), *Weeksellaceae* (Family), *Symbiobacteriaceae* (Family), *Isosphaeraceae* (Family) and *Gemm-5* (Class) were the dominant phyla in bacteria from the 3 SBR reactors, and changes in 5 dominant genera were found for the 3 SBR reactors. The percentages of *Gemmatimonadetes* (Class) in SBR-G1 (Control), SBR-G2 (0.25 g/L) and SBR-G6 (2.0 g/L) were measured at 21.46%, 11.68% and 17.02%, respectively. The percentages of *Weeksellaceae* (Family) in SBR-G1 (Control), SBR-G2 (0.25 g/L) and SBR-G6 (2.0 g/L) were measured at 15.36%, 18.38% and 7.90%, respectively. The percentages of *Symbiobacteriaceae* (Family) in SBR-G1 (Control), SBR-G2 (0.25 g/L) and SBR-G6 (2.0 g/L) were measured at 10.48%, 5.58% and 8.51%, respectively. The percentages of *Isosphaeraceae* (Family) in SBR-G1 (Control), SBR-G2 (0.25 g/L) and SBR-G6 (2.0 g/L) were measured at 5.80%, 11.98% and 15.80%, respectively. The percentages of *Gemm-5* (Class) in SBR-G1 (Control), SBR-G2 (0.25 g/L) and SBR-G6 (2.0 g/L) were measured at 6.82%, 4.16% and 1.42%, respectively. Thus, *Gemmatimonadetes* (Class), *Symbiobacteriaceae* (Family) and *Gemm-5* (Class) were weakened in the Fe_3_O_4_@OMS-2@CTS treatments, while *Isosphaeraceae* (Family) was enriched in the Fe_3_O_4_@OMS-2@CTS treatments. *Weeksellaceae* (Family) was enriched in low concentrations and weakened in a high concentration of Fe_3_O_4_@OMS-2@CTS treatments.

**Figure 4:**
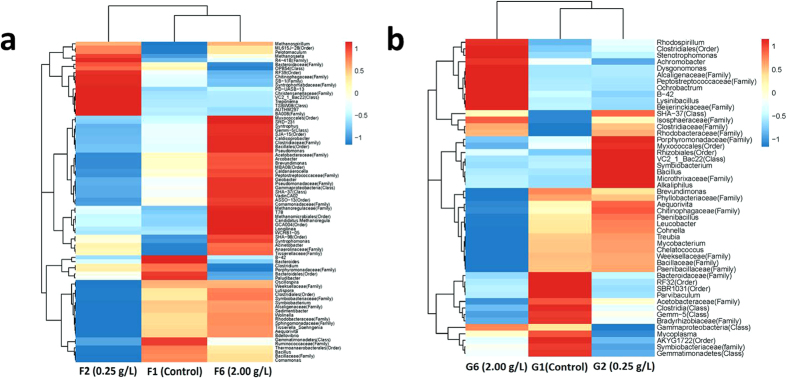
Microbial community heatmap analysis in different treatment. (**a**) Heat-map of the classified genera (abundance ≥ 0.2%) in F1, F2 and F6 UASB reactors; (**b**) Heat-map of the classified genera (abundance ≥ 0.2%) in G1, G2 and G6 SBR reactors.

This microbial community heatmap can be applied to identify differences and similarities between bacterial communities in the UASB and SBR reactors (Fig. 4). As shown in Fig. 4a, 78 bacterial genera in UASB reactors were identified at the 0.2% abundance level. This shows that the bacterial community structures present clear differences in UASB-F1 (Control), UASB-F2 (0.25 g/L) and UASB-F6 (2.0 g/L). The genera were divided into three branches by species richness. From the top to the bottom of Fig. 4a, the first branch covers *Methanospirillum* to *BA008* (Class) with 18 genera, the second branch covers *Myxococcales* (Order) to *Tissierellaceae* (Family) with 34 genera, and the third branch covers *B-42* to *Comamonas* with 26 genera. According to Fig. 4a, the first branch was a high abundance branch in UASB-F2 (0.25 g/L), the second branch was a high abundance branch in UASB-F1 (Control), and the third branch was a high abundance branch in UASB-F6 (2.0 g/L).

From Fig. 4b, 49 bacterial genera in the SBR reactors were identified at the 0.2% abundance level. The results show that bacterial community structures presented clear differences in SBR-G1 (Control), SBR-G2 (0.25 g/L) and SBR-G6 (2.0 g/L). The genera were divided into three branches by species richness. From the top to the bottom of Fig. 4b, the first branch covers *Rhodospirillum* to *Beijerinckiaceae* (Family) with 11 genera; the second branch covers *SHA-37* (Class) to *Paenibacillaceae* (Family) with 25 genera; and the third branch covers *Bacteroidaceae* (Family) to *Gemmatimonadetes* with 13 genera. According to Fig. 4b, the first branch was a high abundance branch in SBR-G6 (2.0 g/L), the second branch was a high abundance branch in SBR-G2 (0.25 g/L) and the third branch was a high abundance branch in SBR-G1 (Control).

### Mechanism discussion

According to the previously described results, adding Fe_3_O_4_@OMS-2@CTS can affect UASB and SBR reactors, generally leading to enhanced X-3B decolourization ratio (Table 1 and Fig. 2). This is due to the microbial community in UASB and SBR changing after addition of Fe_3_O_4_@OMS-2 (Figs 3 and 4), i.e., a higher microbial diversity (Fig. 3) and change of predominant microorganisms in the reactors. Specifically, (1) the competing bacteria (e.g., *Firmicutes*, *Bacteroidetes*, and *Proteobacteria* in the UASB reactor; *Gemmatimonadetes* in the SBR reactor) were inhibited, contributing to the increase of microbial diversity; (2) the abundance of *Chloroflexi* bacteria in UASB reactor was enhanced, which promoted anaerobic degradation of wastewater^27^; (3) the increase of *Proteobacteria* bacteria in SBR reactor enhanced the biological removal of organic pollutants^30^ and *Proteobacteria* was used as a sign of dysbiosis in bacterial communities^29^; and (4) the abundance of *Bacteroidetes* bacteria was enriched in low concentrations and weakened in high concentrations of Fe_3_O_4_@OMS-2 @CTS treatments in the SBR reactor; the increase of *Bacteroidetes* bacteria could correlate with alleviation of metabolic disorders and promote microbial metabolism^28^. Compared to the control test without addition of material, this change on microbial community must be attributed to the NPs, which include: (1) NPs can incorporate into the mixed liquor suspended solids (MLSS) (containing the microbial community) and then change the microbial community; (2) bacteria could easily attach onto the microspheres of chitosan, and the effect of Fe_3_O_4_@OMS-2 would be enhanced; and (3) Fe and Mn originating from NPs could interact with the microbe, resulting in a reduction of Fe(III) and Mn(IV). Specifically, adding Fe_3_O_4_@OMS-2@CTS can enhance the decolourization ratio (not more than 0.50 g/L) and decrease it at high concentrations (more than 0.50 g/L and not more than 2.00 g/L) in UASB reactors. However, adding Fe_3_O_4_@OMS-2@CTS can enhance the decolourization ratio (not more than 2.00 g/L). This change in UASB and SBR reactors may result from the modulation of Fe_3_O_4_@OMS-2@CTS, which alters microbial communities in UASB and SBR reactors (Figs 3 and 4).

**Figure 5:**
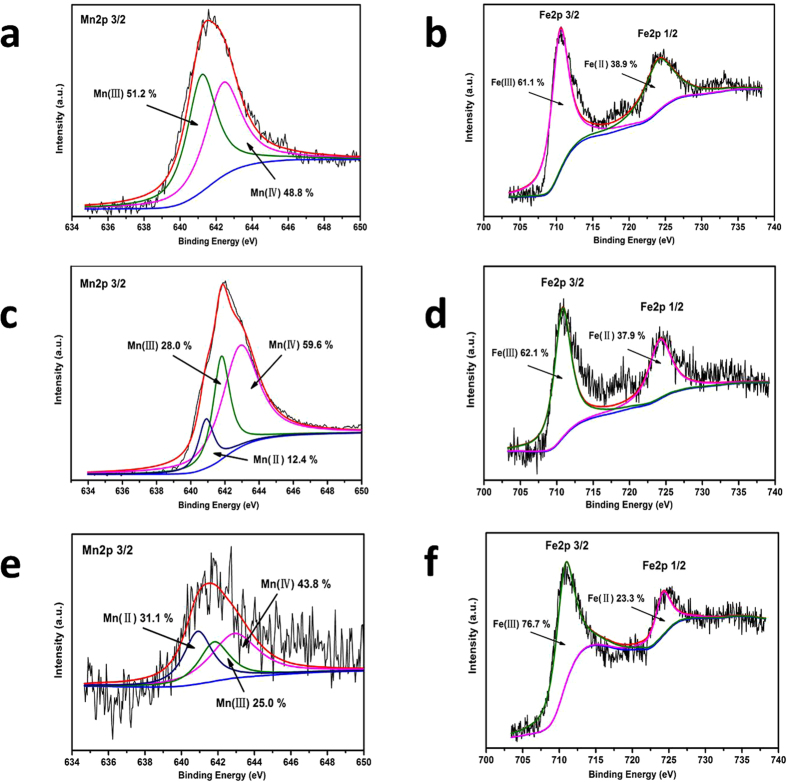
Changes of Fe_3_O_4_@OMS-2@CTS before and after in different treatments: (**a**) XPS spectra of Mn 2p of fresh Fe_3_O_4_@OMS-2@CTS; (**b**) XPS spectra of Fe 2p of fresh Fe_3_O_4_@OMS-2 @CTS; (**c**) XPS spectra of Mn 2p of used Fe_3_O_4_@OMS-2@CTS in UASB reactors; (**d**) XPS spectra of Fe 2p of used Fe_3_O_4_@OMS-2@CTS in UASB reactors; (**e**) XPS spectra of Mn 2p of used Fe_3_O_4_@OMS-2 @CTS in SBR reactors; and (**f**) XPS spectra of Fe 2p of used Fe_3_O_4_@OMS-2@CTS in SBR reactors.

Fe_3_O_4_@OMS-2@CTS contains different oxidation states of manganese (e.g., Mn(IV), Mn(III) and Mn(II)) and oxidation states of iron (e.g., Fe(III) and Fe(II)). Microorganisms have the capacity for dissimilatory Fe(III) and Mn(IV) reduction, which involves transferring electrons to Fe(III) and Mn(IV)^32^. In addition, considering the extremely low concentrations of background Fe (0.5 μg/L) and Mn (1.88 μg/L) in the synthetic wastewater, Fe and Mn from NPs played a dominant role after material addition. Mn(III)/Mn(IV) oxide and Fe(III) oxide are relatively insoluble and stable, while Mn(II) oxide and Fe(II) oxide can be solubilized under acidic conditions. To investigate changes in Fe and Mn species in the Fe_3_O_4_@OMS-2@CTS, XPS spectra of Fe_3_O_4_@OMS-2@CTS were measured before and after the UASB and SBR experiments were conducted. For the Mn 2p_3/2_ spectra of Fe_3_O_4_@OMS-2@CTS, two peaks at 642.4 and 641.2 eV with a peak area ratio of 48.8:51.2 were found (Fig. 5a), and these may be designated Mn(III) and Mn(IV) oxidation states, respectively. The XPS spectra of Fe 2p are shown in Fig. 5b. We found two peaks centred at 710 and 724 eV indexed to Fe 2p_3/2_ and Fe 2p_1/2_ with a ratio of 61.1:38.9, which are consistent with the Fe(III) and Fe(II) oxidation states, respectively^33^.

After the UASB experiments were conducted, the third peak accredited to the Mn(II) oxidation states was identified in Fe_3_O_4_@OMS-2@CTS, and its atomic ratio was measured at 59.6:28.0:12.4 (Mn(IV): Mn(III): Mn(II)) (Fig. 5c). The atomic ratio of Fe(III) and Fe(II) in Fe_3_O_4_@OMS-2@CTS was measured at 62.1:37.9 (Fe(III): Fe(II)) (Fig. 5d) in UASB reactor, indicating almost no change of Fe states. However, after the SBR experiments were conducted, a third peak accredited to the Mn(II) oxidation states was identified in Fe_3_O_4_@OMS-2@CTS with an atomic ratio of 43.8:25.0:31.1 (Mn(IV): Mn(III): Mn(II)) (Fig. 5e). The atomic ratio of Fe(III) and Fe(II) in Fe_3_O_4_@OMS-2@CTS was measured at 76.7:23.3(Fe(III): Fe(II)) (Fig. 5f). The much higher Fe(III) ratio in SBR reactor was due to oxidation of Fe(II) by aeration, and interaction of Fe(II) with microorganism or metabolites.

The XPS results show that Mn(IV)/Mn(III) with redox were reduced to Mn(II) in both the UASB and SBR reactors, but Mn(IV)/Mn(III) with redox in the UASB reactors was less than that found in the SBR reactors. The XPS results also show a very small change in oxidation states of Fe(III)/Fe(II) in Fe_3_O_4_@OMS-2@CTS in the UASB reactors (only 2%) but a significant change in oxidation states of Fe(III)/Fe(II) in Fe_3_O_4_@OMS-2@CTS in the SBR reactors. Moreover, we should notice that XPS can only perform a surface analysis of the material; other characterizations such as X-ray absorption spectroscopy (XAS), extended X-ray absorption fine structure (EXAFS) and electron paramagnetic resonance (EPR) can be further conducted to deeply investigate the change of Mn/Fe during the reaction.

Mn(IV)/Mn(III) and Fe(III)/Fe(II) with redox offered reducing equivalents to the microorganisms and a broad variety of microorganisms can acquire energy from Mn(IV)/Mn(III) with redox. Emily *et al*. found that microorganisms, which were discovered from marine sediment in California, could utilize Mn(IV) and Fe(III) to oxidize methane and proved that Mn(IV) and Fe(III) could be used as electron acceptors in anaerobic oxidation of methane (AOM)^34^. Mukhopadhyay *et al*. reported that metal manganese blocked intracellular trafficking of Shiga toxin (STx) and affected its degradation in lysosome^35^.

Generally, the dissimilatory Mn(IV)-reducing microorganisms contain two major groups: (1) Mn(IV)-respiring microorganisms (FMR), which support growth by conserving energy from electron transfer to Mn(IV)^5^; and (2) non-Mn(IV)-respiring microorganisms (Non-FMR). FMR are widely dispersed throughout *Bacteria* and *Archaea*, which grow by oxidizing organic compounds, hydrogen or S^0^ associated with a reduction of Mn(IV)^32^. In this study, the addition of Fe_3_O_4_@OMS-2@CTS into the UASB and SBR reactors can promote FMR growth, such as *Chloroflexi* bacteria in UASB and *Proteobacteria* bacteria in SBR (Fig. 3). On the other hand, the terminal electron-accepting process (TEAP) of Mn(IV) reduction also occur in the non-FMR metabolic process, although they do not gain energy from the dissimilatory reduction of Mn(IV), e.g., *Lactococcus spp*^36^. Moreover, widespread reoxidation processes of Fe(II) are due to its biological utilization as an electron donor by aerobic bacteria^37^. Fe(II) in microorganisms plays a role in several key enzymatic activity, e.g., pyruvate-ferredoxin oxidoreductase, which plays a major role in fermentation^38^. Byrne *et al*. discovered that some microorganisms had the ability of utilization both Fe(III) and Fe(II) for growth and believed that Fe ions bound could be bioavailable as electron sources and electron sinks under different environment conditions. For example, *Rhodopseudomonas palustris* TIE-1 could oxidize magnetite nanoparticles by using light energy, while *Geobacter sulfurreducens* could reverse the process in co-cultures^39^. Microorganisms of both heterotrophic and autotrophic groups can turn nitrate into an electron acceptor^40^,^41^.

## Conclusions

This study evaluated the effects of manganese oxide octahedral molecular sieve chitosan microspheres (Fe_3_O_4_@OMS-2@CTS) on anaerobic and aerobic microbial communities in typical sewage biological treatments, i.e., UASB and SBR. The decolourization ratio changes in the UASB reactors were found to be different from those observed in the SBR reactors. The microbial community changes in the UASB reactors were also different from those observed in the SBR reactors. Mn(IV)/Mn(III) and Fe(III)/Fe(II) with great redox were found in sewage biological treatments. This study provides basic information on the effect of nanoparticles on contaminant removal and microbial community change in the conventional biological wastewater treatment process. In the future, comparison on contaminants removal by pure nanoprocess, pure biological process, and the nanomaterial-enhanced biological process can be further investigated.

## Methods

### Preparation of Fe_3_O_4_@OMS-2@CTS catalyst

Fe_3_O_4_@OMS-2 was synthesized using a reflux method that is similar to a procedure reported elsewhere^16^. Mannich reaction methods were used to prepare the chitosan grafted ligand^19^. Further information on the characterization and synthesis of Fe_3_O_4_@OMS-2@CTS is provided in Text S1 (Supplementary Information).

### Experimental setup

Six groups of anaerobic reactors (F1-F6) and six groups of aerobic reactors (G1-G6) were employed. The F1 of the anaerobic reactors and the G1 of the aerobic reactors formed the control group without the addition of Fe_3_O_4_@OMS-2@CTS. F2-F6 and G2-G6 formed the treatment groups with the addition of variable concentrations of Fe_3_O_4_@OMS-2@CTS. PMS (2KHSO_5_•KHSO_4_•K_2_SO_4_) was added to both the UASB and SBR operation and the dose of PMS was 0.125 g/L. Figure 1 presents a schematic diagram of the anaerobic and aerobic reactors. The anaerobic reactor was an upflow anaerobic sludge blanket (UASB), and the aerobic reactor employed a sequencing batch reactor activated sludge process (SBR). The parameters of UASB reactors and SBR reactors were listed in Text S2 (Supplementary Information).

### Inoculated sludge and synthetic dyeing wastewater

Inoculated sludge was drawn from anaerobic and aerobic digesters of the sewage treatment plant based at Wuhan Textile University (Wuhan, China). The inoculated sludge was acclimated for 35 d to allow it to degrade X-3B dye. The inoculated sludge had a pH level of 6.5–7.2. The inoculated sludge included 4098 mg/L of mixed liquor suspended solids (MLSS) and 2665 mg/L of mixed liquor volatile suspended solids (MLVSS). The influent dyeing wastewater was synthesized with X-3B dye using the following stock solution: NH_4_HCO_3_ 14.1 g/L, sucrose 90 g/L, K_2_HPO_4_•3H_2_O 1.62 g/L, KH_2_PO_4_ 0.97 g/L and NaHCO_3_ 33.3 g/L. The chemical oxygen demand (COD) level of the stock dyeing wastewater was measured at approximately 100 g/L. The desired COD concentration was then diluted with ingredients that had the following trace elements: CuSO_4_·5H_2_O 58, H_3_BO_3_ 100, ZnSO_4_·7H_2_O 100, FeSO_4_·7H_2_O 750, NiSO_4_·6H_2_O 112, MnSO_4_·H_2_O 188, MgSO_4_·7H_2_O 5000, NaMo_7_O_24_·2H_2_O, 50, FeSO_4_·7H_2_O 50, CaCl_2_·2H_2_O 7500, and yeast 3000 μg/L. These were added before the stock dyeing wastewater was prepared for experimentation.

## Analytical Methods

### Degradation of reactive brilliant red X-3B by Fe_3_O_4_@OMS-2@CTS /PMS

All of the catalytic degradation experiments were performed in a 100-mL reactor at approximately 30 °C. Fe_3_O_4_@OMS-2@CTS was added to the reactor after adding desired amounts of X-3B and PMS (2KHSO_5_•KHSO_4_•K_2_SO_4_, Aladdin) in 50 mL of the aqueous solution. The reaction solution was repeatedly stirred with a magnetic stirrer that was coated with PTFE. Further information on degradation of X-3B by Fe_3_O_4_@OMS-2@CTS/PMS is listed in Text S3 and Fig. S6 (Supplementary Information).

### Degradation of reactive brilliant red X-3B by Fe_3_O_4_@OMS-2@CTS /PMS during sewage biological treatment

The UASB reactors were first operated with COD of synthetic dyeing wastewater loading from 100 to 4,000 g/m•d for two months for the pre-acclimation of anaerobic microbiology. The COD values of both influents and effluents in all of the UASB reactors were monitored using standard methods. The UASB reactors were then operated over 4 phases, i.e., start-up (0–72 d), X-3B loading increase (73–121 d), recovery (122–140 d) and stable operation (141–219 d). On Day 149, Fe_3_O_4_@OMS-2@CTS of 0.25, 0.50, 1.00, 1.50 and 2.00 g/L were added to the F2–F6 reactors, and F1 served as the control group wherein no Fe_3_O_4_@OMS-2@CTS was spiked. The experimental schedule for COD and X-3B loadings of UASB is shown in Table S1 of the Supplementary Information section. The operation temperature of the UASB reactors was maintained at (30 ± 2 °C), the hydraulic retention time (HRT) was set to 18 ± 2 h, and the average cell residence time ranged from 20 to 40 d. On Day 209, samples were collected from the 6 groups of UASB reactors.

Freshly activated sludge was inoculated at a concentration of 4,000 ± 81 mg/L (MLSS) in the 6 SBR reactors. Fe_3_O_4_@OMS-2@CTS volumes of 0.25, 0.50, 1.00, 1.50 and 2.00 g/L combined with PMS (0.125 g/L) were also added to G2–G6 in the SBR reactors, with G1 serving as the control group wherein no Fe_3_O_4_@OMS-2@CTS was spiked. The detailed operating parameters for the 6 SBR reactors are listed in Table S2 of the Supplementary Information section.

Synthetic X-3B dyeing wastewater was continuously pumped through the SBR column. The flow rate of the SBR influent was set at 2.8 ± 0.1 mL/min. The 6 SBR reactors (G1–G6) were operated sequentially over 12 h cycles that consisted of 11.5 h of aeration time, 10 min of influent filling, 10 min of effluent withdrawal and 10 min of settling. The hydraulic residence time (HRT) was set to 20 h, and the exchange volume ratio discharged from the SBR reactors was set to 60%. There was a bubble aerator in the bottom of the SBR column, and the air flow rate was set to 1.5 L/min. The operating temperature of all of the SBR reactors was set to 30 ± 2 °C. On Day 60, the samples were collected from the SBR reactor.

The morphology characterization of Fe_3_O_4_@OMS-2@CTS in the UASB and SBR reactors was subjected to SU8020 Ultra-High-Resolution FE-SEM (Hitachi, Tokyo, Japan). The X-ray photoelectron spectroscopy of Fe_3_O_4_@OMS-2@CTS was conducted using a Thermo ESCALAB 250XI Multifunctional imaging electron spectrometer (Thermo Fisher Scientific, Waltham, USA). The anaerobic and aerobic microorganism genera were identified via Illumina MiSeq high-throughput sequencing.

### Illumina high-throughput sequencing

The microbial DNA of liquor samples drawn from the anaerobic and aerobic reactors was isolated using an E.Z.N.A. Soil DNA Kit (Omega Bio-Tek, Norcross, GA, USA). The microbial DNA extracts were then stored at −20 °C for PCR amplification. The universal V4 regions of the 16 S rRNA genes primers were set to 520 F (5′-AYTGGGYDTAAAGNG-3′) and 802 R (5′-TACNVGGGTATCTAATCC-3′)^5^. The extracted DNA barcode and adapter with tags were added between the adapters and forward primers. Detailed information on PCR amplification is shown in Text S4 of Supplementary Information. The amplicons of PCR were then detected by 0.8% agarose gels with a loading level of 3 μL. The electrophoresis results of all of the amplicons were satisfactory with clear strap (Fig. S7, Supplementary Information). The multiplexed DNA libraries were homogenized to 10 nM and mixed at equal volumes. The mixed DNA libraries were then progressively and quantitatively diluted to 4–5 pM and then subjected to paired-end sequencing (2 × 250) on an Illumina MiSeq platform.

The obtained raw fastq were demultiplexed and quality-filtered via Qiime (version 1.7.0, http://qiime.org/) and Mothur (version 1.31.2, http: //www. mothur. org/) to obtain high-quality sequences for subsequent analysis. Operational Taxonomic Units (OTUs) were also clustered with 97% similarity using Qiime software.

### Statistical analysis

A bicluster analysis of high-throughput sequencings was conducted using the pheatmap package available through the R software program. Statistical analysis was performed using Origin 8.1 and SPSS 19.0. Statistical significance was designated at a value of *P* < 0.05.

## Additional Information

**How to cite this article**: Pan, F. *et al*. The effects of manganese oxide octahedral molecular sieve chitosan microspheres on sludge bacterial community structures during sewage biological treatment. *Sci. Rep*. **6**, 37518; doi: 10.1038/srep37518 (2016).

**Publisher’s note:** Springer Nature remains neutral with regard to jurisdictional claims in published maps and institutional affiliations.

## Supplementary Material

Supplementary Information

## Figures and Tables

**Table 1:** COD removal efficiency, decolourization ratio and COD loading and effluent in different treatments.

	COD removal efficiency (%)	Decolourization ratio (%)	COD loading (g/L•d)	COD effluent (g/L•d)
F1 (Control)	87.61 ± 1.39	60.09 ± 0.81	4.0	0.49
F2 (0.25 g/L)	89.36 ± 1.43	64.63 ± 0.97	4.0	0.42
F3 (0.50 g/L)	91.79 ± 1.52	66.32 ± 0.93	4.0	0.33
F4 (1.00 g/L)	86.85 ± 1.36	58.02 ± 0.72	4.0	0.52
F5 (1.50 g/L)	86.41 ± 1.15	55.99 ± 0.81	4.0	0.54
F6 (2.00 g/L)	85.73 ± 1.26	56.51 ± 0.75	4.0	0.56
G1 (Control)	78.53 ± 1.27	79.10 ± 1.16	1.2	0.25
G2 (0.25 g/L)	85.82 ± 1.37	95.21 ± 1.28	1.2	0.16
G3 (0.50 g/L)	86.37 ± 1.32	95.30 ± 1.31	1.2	0.15
G4 (1.00 g/L)	84.54 ± 1.24	93.10 ± 1.42	1.2	0.18
G5 (1.50 g/L)	83.73 ± 1.21	84.81 ± 1.24	1.2	0.19
G6 (2.00 g/L)	82.31 ± 1.13	83.98 ± 1.19	1.2	0.20

**Table 2:** Different microbial diversity indices in different treatments.

Sample	Valid Sequence	Chao1	Ace	Simpson	Shannon
F1 (Control)	91102	1253	1282	0.066	4.005
F2 (0.25 g/L)	73983	1259	1289	0.058	4.067
F6 (2.00 g/L)	106594	1248	1254	0.048	4.061
G1 (Control)	97829	720	738	0.083	3.400
G2 (0.25 g/L)	83451	746	756	0.064	3.621
G6 (2.00 g/L)	81218	727	753	0.066	3.496
