# Supplementary material for: The effects of manganese oxide octahedral molecular sieve chitosan microspheres on sludge bacterial community structures during sewage biological treatment
Source: Sci Rep. 2016 Nov 21;6:37518. doi: 10.1038/srep37518 (PMC5116614; doi:10.1038/srep37518)
Supplement: Supplementary Information [file srep37518-s1.pdf]

Supplementary Information for

The effects of manganese oxide octahedral molecular sieve chitosan microspheres on sludge bacterial community structures during sewage biological treatment

Fei Pan[1,2,3,*], Wen Liu[3], Yang Yu[1], Xianze Yin[4], Qingrong Wang[1], Ziyan Zheng[1], Min Wu[1], Dongye Zhao[3], Qiu Zhang[1], Xiaoman Lei[1], Dongsheng Xia[1,*]

[1] School of Environmental Engineering, Wuhan Textile University, Wuhan 430073, China

[2] Engineering Research Centre for Clean Production of Textile Dyeing and Printing, Ministry of Education, Wuhan Textile University, Wuhan 430073, China

[3] Environmental Engineering Program, Department of Civil Engineering, Auburn University, Auburn, AL 36849, USA

[4] School of Materials Science and Engineering, Wuhan Textile University, Wuhan 430073, China

[*] **Corresponding authors**: Address: 1 Fangzhi Road, School of Environmental Engineering, Wuhan Textile University, Wuhan, 430073, China. Tel. & Fax: +86-027-59367338. E-mail address: leopan0724@189.cn (F. Pan). Or E-mail address: xds01@163.com (D.S. Xia).

Text S1: Characterization and synthesis of Fe_3O_4@OMS-2@CTS

OMS-2 was synthesized by a reflux method, which was previously reported.[1] In brief, 3 mL nitric acid and 60 mmol manganese sulfate hydrate ($MnSO_4$) were added in 30 mL double deionized water (DDW). And another solution which dissolving 38 mmol $KMnO_4$ in 100 DDW was prepared. The second solution was successively added dropwise into the $MnSO_4$ solution under intense stirring at 110 °C to form dark brown sediment. The precipitate was heated overnight at the reflux temperature, then washed it with DDW, and dried at 120 °C for 12 h at last to obtain OMS-2.

Magnetic chitosan microsphere was synthesized by a reflux method, which was previously reported[2]. In brief, 0.5 g chitosan was dissolved in 5 % aqueous acetic acid solution; The second solution was added dry OMS-2 and nano-Fe_3O_4 into the chitosan solution at room temperature to ultrasonic vibration for 40 min; And the dispersion medium was prepared by composing mineral oil and petroleum with 25:35 (*v/v*), and Tween-80; then it was poured by dropwise into the dispersion medium under vigorous stirring at 1000 -2000 rpm for 10 min. then 1 mL glutaraldehyde was added into the medium and another 1 mL glutaraldehyde was added into the dispersion medium after 1 h later, then the dispersion medium was stirred for 2 h.

In the next step, the magnetic chitosan microspheres were gathered by using a magnet, and were washed in succession with petroleum ether, sodium bisulfide, and acetone. In the end, the magnetic chitosan microspheres were dried in an oven at 40

°C for 48 h and kept in a vacuum dessicator for use.

The X-ray diffraction (XRD) pattern of Fe_3O_4@OMS-2@CTS was obtained on an Thermo ESCALAB 250XI Multifunctional imaging electron spectrometer (Thermo Fisher Scientific, Waltham, USA),and the result is shown in Figure S4a. All peaks can be indexed to pure cryptomelane phase (JCPDS 29-1020). By using the Scherrer equation for the (211) peak, the size for OMS-2 on Fe_3O_4 was calculated to be 7.3 nm.

The FT-IR pattern of Fe_3O_4@OMS-2@CTS was obtained on a Tensor 27X FTIR spectrometer (Bruker, Billerica, MA, USA), and the result is shown in Figure S4b. The OH group present in the chitosan polymer is seen at 3446 cm^{-1} and the OH group present in Fe_3O_4@OMS-2@CTS is 3450 cm^{-1} and the similar intensities are also present in the case of microsphere forms. Furthermore the glutaraldehyde cross-linking was shown by the carbonyl bands at around 1700 cm^{-1} in the case of the microsphere forms.

The Mn and Fe oxidation states in Fe_3O_4@OMS-2@CTS before and after reaction were analysed by X-ray photoelectron spectra (XPS) on a multifunctional imaging electron spectrometer (Thermo Scientific ESCALAB 250Xi) with Al *K*a radiation as the exciting source (150 W). Charging effects were corrected by adjusting the binding energy of C 1s to 284.6 eV.

For all the cases with different addition of Fe_3O_4@OMS-2@CTS, the concentration of dissolved Mn ions in the aqueous phase was determined on an inductively coupled plasma-mass (ICP-MS, X Series II, Thermo Fisher Scientific, USA)

after the sample was filtered through a 0.22 μm membrane. The total dissolved Mn in solution was found <1 % for all cases.

Text S2: The parameters of UASB reactors and SBR reactors

The anaerobic reactor was an upflow anaerobic sludge blanket (UASB) composed of a plexiglass column. The UASB reactor had an internal diameter of 70 mm and an overall height of 500 mm. The UASB reactor was equipped with a 1.8-L effective volume, and a three-phase separator was fitted on top of the UASB reactor. The aerobic reactor employed a sequencing batch reactor activated sludge process (SBR) that also involved the use of a plexiglass column. The SBR reactor had an internal diameter of 100 mm and an overall height of 300 mm. The SBR reactor had an effective volume of 2.3 L, and an aeration device was fitted to the base of SBR reactor. The aeration device was attached by a silicone tube fitted with a pump to increase dissolved oxygen levels in wastewater in the SBR reactor. Wastewater entered the UASB reactor from the bottom via a peristaltic pump at a liquid upflow speed of 1.0 m/h, and the gas produced escaped from an upper tube separated by the three-phase separator at the anaerobic reactor. Dissolved wastewater oxygen in the SBR reactor was maintained at 3 mg/L by an aeration pump.

The operation temperature of the UASB reactors was maintained at (30 ± 2 °C), the hydraulic retention time (HRT) ranged from 10 to 20 h (close to 20 h), and the average cell residence time ranged from 20 to 40 d. We chose the double average cell residence time (60 days) as the reaction time of the Fe_3O_4@OMS-2@CTS and then the samples were collected on 60th day from the 6 groups of UASB reactors. The Fe_3O_4@OMS-2@CTS in SBR reactors were reaction for the same length of time and also collected on 60th day.

Text S3: Degradation X-3B by Fe_3O_4@OMS-2@CTS/PMS

For studying the effect of the initial concentration of Fe_3O_4@OMS-2@CTS /PMS on the rate of X-3B degradation, X-3B (50 mg/L) and PMS (0.25 g/L) were added into the solution and the solution pH was adjusted at 3.58 ± 0.2 by H_2SO_4 (0.1 mol/L). The range of initial concentrations of Fe_3O_4@OMS-2@CTS/PMS in the solution was 0.25 g/L to 2.0 g/L. To monitor the degradation of X-3B, the solution samples were measured on a UV-vis spectrophotometer (UV1100, Beijing Rayleigh analytical instrument Corp., Beijing, China) under the maximum absorption wavelength (540 nm) at given time intervals. And the curve of degradation X-3B by Fe_3O_4@OMS-2 @CTS/PMS was shown in Fig.S6.

Text S4: The detailed information on PCR amplification

The PCR mixture contained 20 ng of DNA temple, 2.00 µL of 2.5 mM dNTPs, 1.00 µL of Forward primers, 1.00 µL of Reverse primers, 0.25 µL of 5U Q5 Polymerase, 5.0 µL of 5 × Q5 GC high Enhancer and 5.0 µL of 5 × Q5 Reaction Buffer. Sterile double-distilled H_2O was performed via PCR amplification at a total volume of 25 µL. The PCR protocol involved an initial 30 s denaturation at 98 °C followed by 25 cycles of denaturing at 98 °C for 30 s, annealing at 50 °C for 30 s, extension at 72 °C for 30 s, and final extension at 72 °C for 5 min.

References:

1. Pan, F. *et al.* Effects of octahedral molecular sieve on treatment performance, microbial metabolism, and microbial community in expanded granular sludge bed reactor. *Water Res.* **87**, 127-136 (2015).
2. Denkbaş, E. B., Kiliçay, E., Birlikseven, C. & Öztürk, E. Magnetic chitosan microspheres: preparation and characterization. *React. Funct. Polym.* **50**, 225-232 (2002).

Table S1. The experimental schedule for COD and X-3B loadings of UASB

Phase	Time (d)	COD (g/m^3•d)	X-3B
		F1 - F6	F1 - F6
**1:Start-up**	0-72	4000 ± 48	2.6-25.1
**2:X-3B loading increase**	73-121	7000 ± 79	26.7-60.3
**3:Recovery**	122-140	3000 ± 21	16.5-30.4
**4:Stable operation**	141-219	4000 ± 42	25.7-27.3

Table S2. Operating parameters for the 6 SBR reactors

Operational parameter	SBR (G1 - G6)
**Hydraulic retention time /h**	20
**Cycle time /h**	12
**Exchange time /h**	0.5
**Aeration time /h**	11.5
**Air flow rate /L•min^{-1}**	1.5
**Exchange volume ratio/%**	60
**Sludge retention time /d**	5
**pH of influent**	6.5 – 7.2

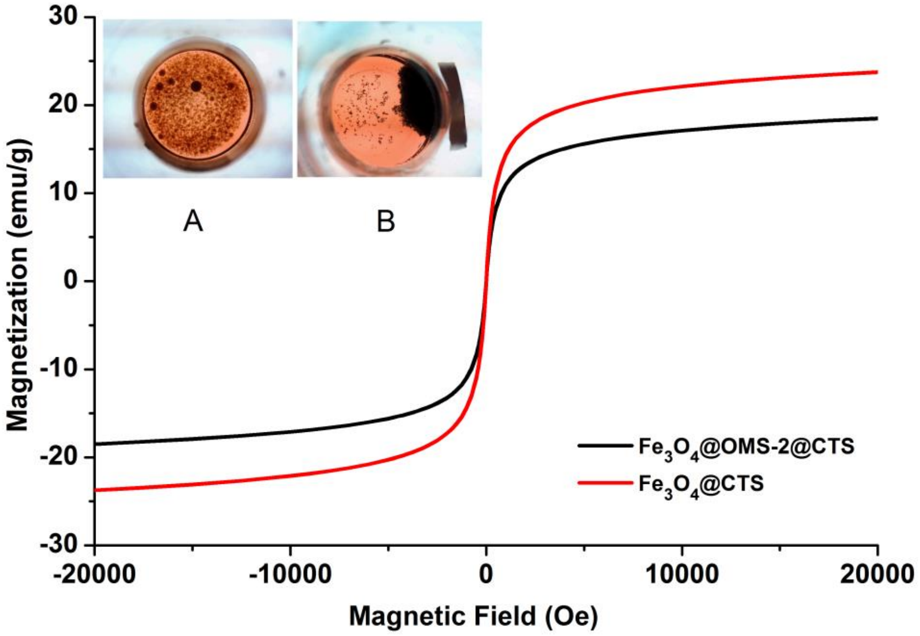

Figure S1. *M-H* hysteresis loops of Fe_3O_4@OMS-2@CTS. Inset is the photographs of Fe_3O_4@OMS-2@CTS in dyeing wastewater.

(a)

1-1’

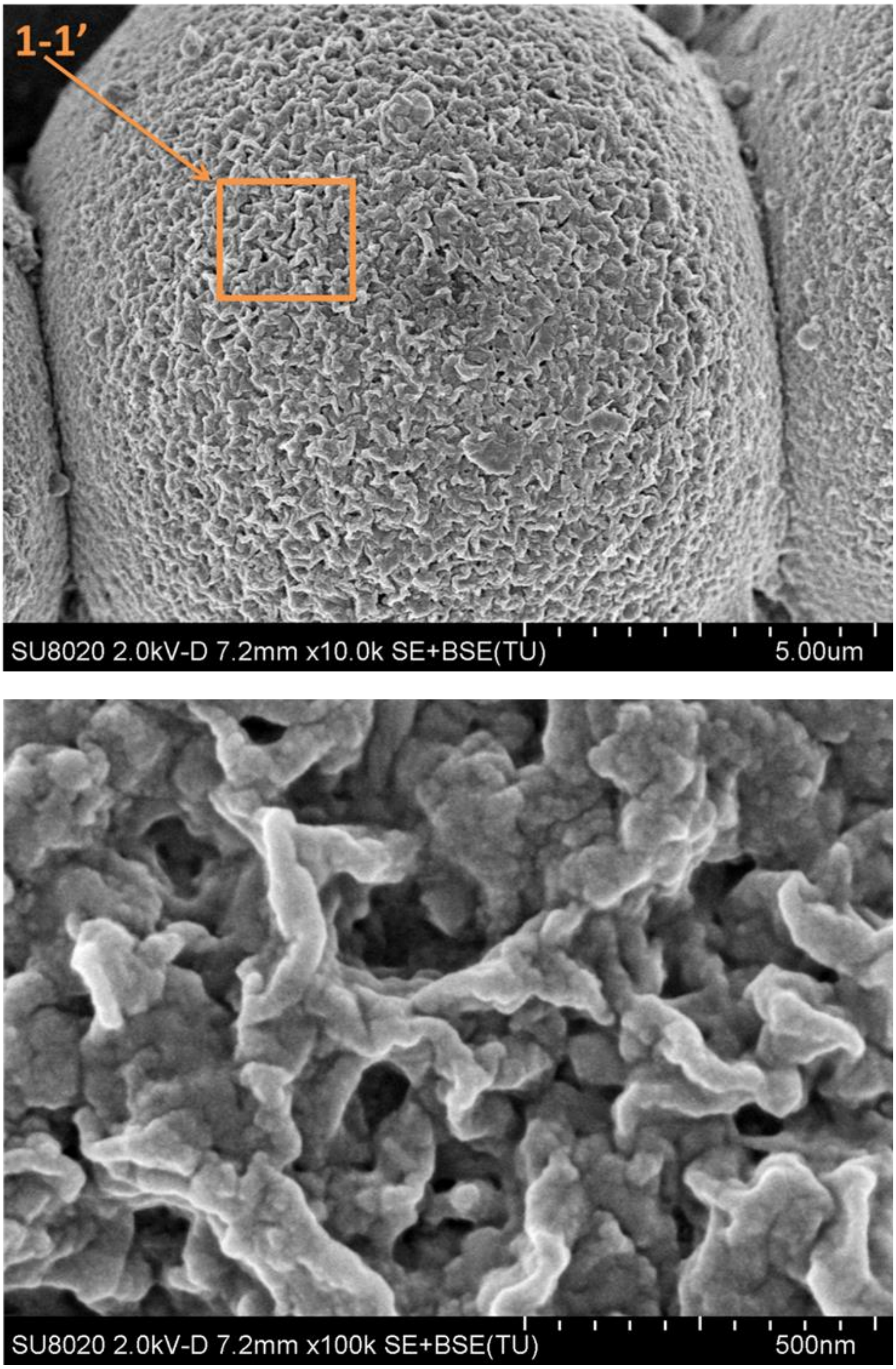

Figure S2. The whole surface (a) and the section (1-1’) of fresh Fe_3O_4@OMS-2@CTS.

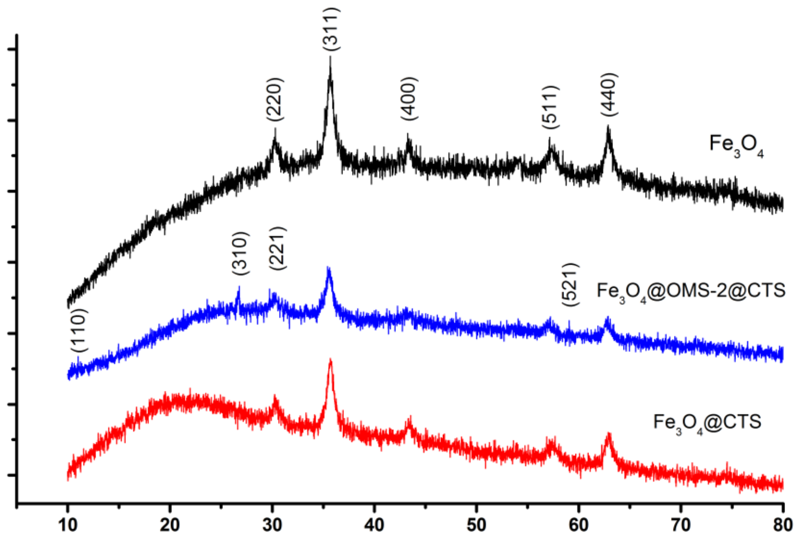

(a)

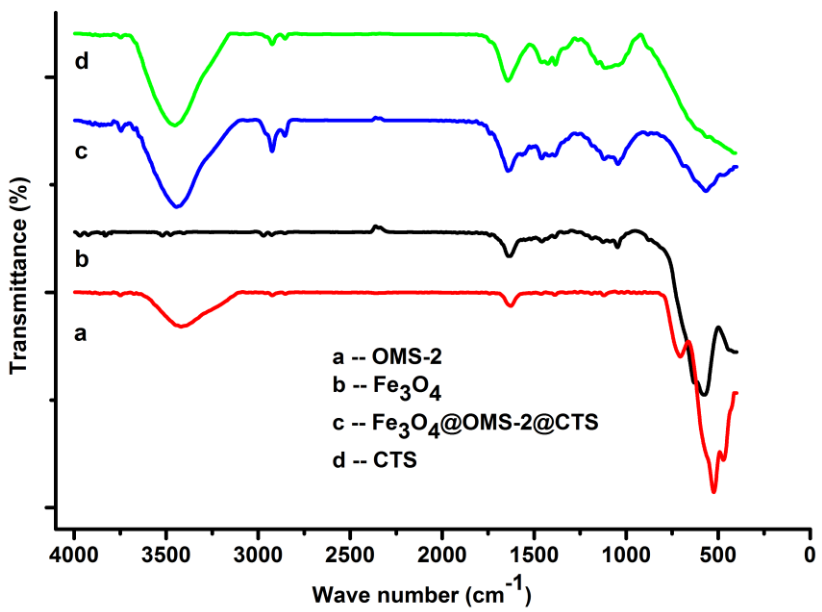

(b)

Figure S3. (a) XRD profile and **(b)** FT-IR spectroscopy of Fe_3O_4@OMS-2@CTS.

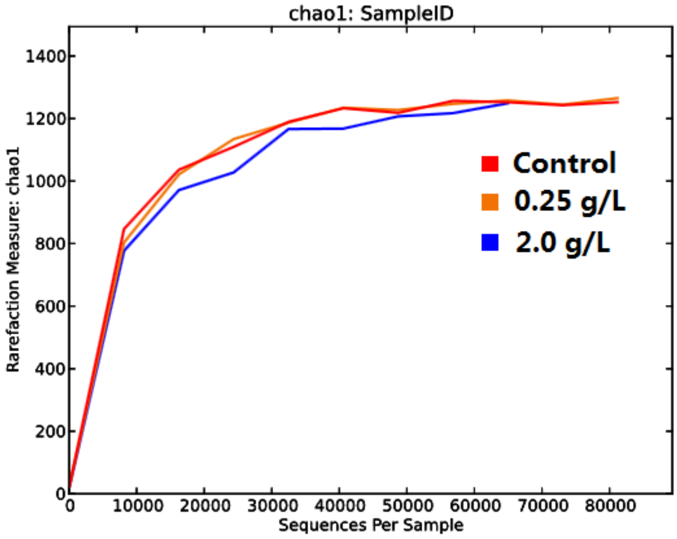

(a)

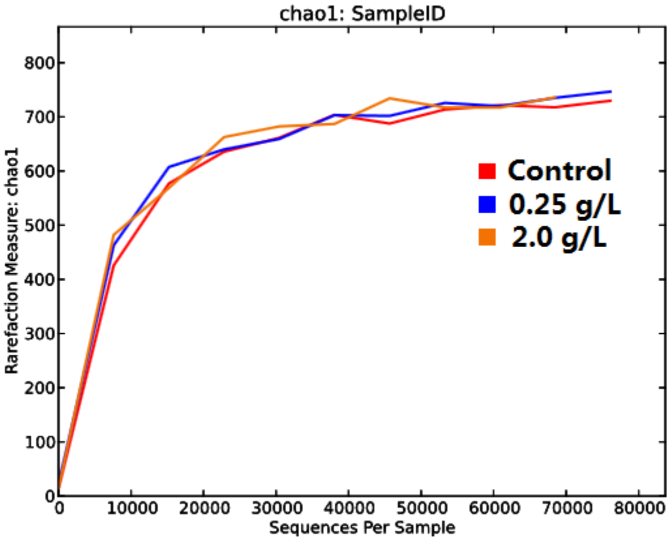

(b)

Figure S4. The rarefaction curves of samples in UASB **(a)** and SBR **(b)** reactors

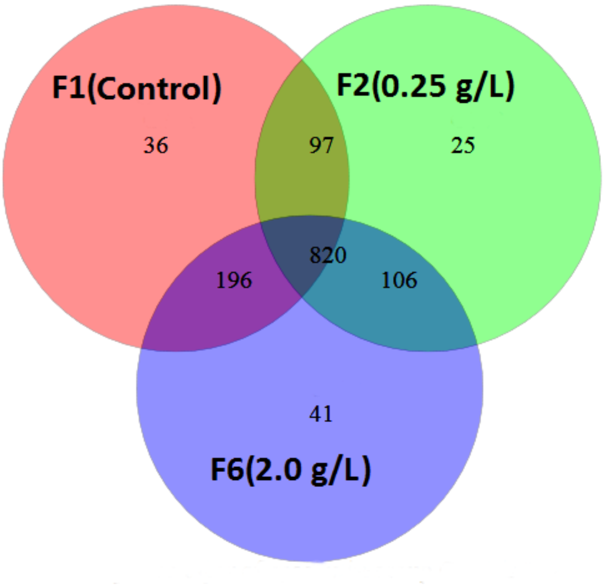

(a)

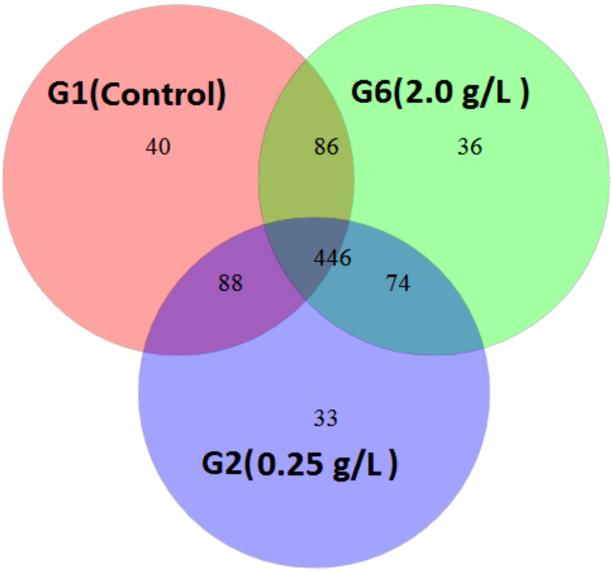

(b)

Figure S5. (a) Venn diagram of OTUs in F1, F2 and F6 samples;
(b) Venn diagram of OTUs in G1, G2 and G6 samples

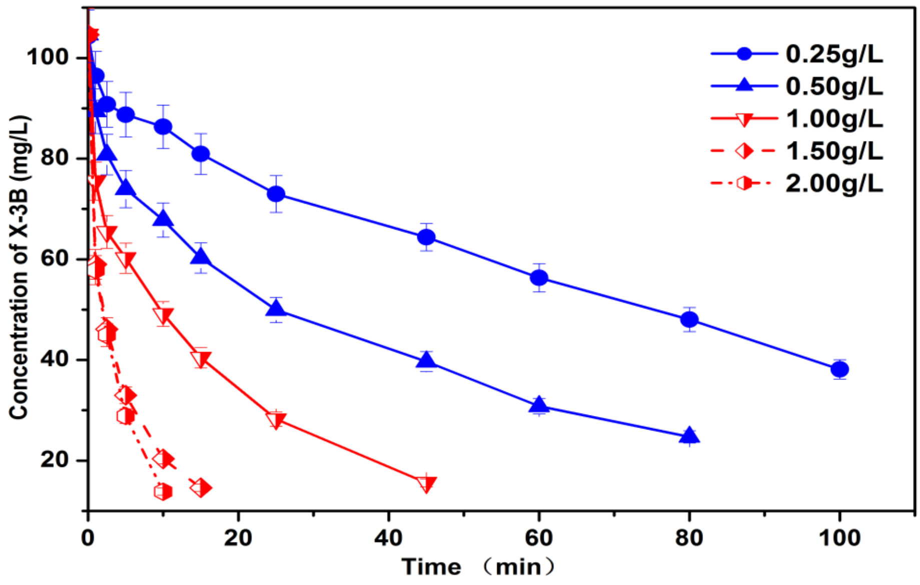

Figure S6. The curve of degradation X-3B by Fe_3O_4@OMS-2@CTS/PMS

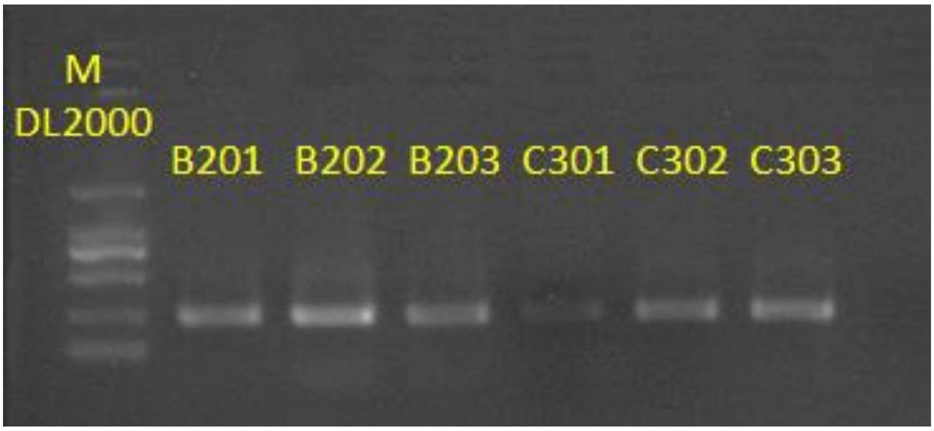

Figure S7. The electrophoresis result of all the amplicons

B201: F1 (Control), B202: F2 (0.25 g/L), B203:F6 (2.0 g/L);

C301: G1 (Control), C302:G2 (0.25 g/L), C303:G6 (2.0 g/L);
